# Randomized trial of primaquine hypnozoitocidal efficacy when administered with artemisinin-combined blood schizontocides for radical cure of *Plasmodium vivax* in Indonesia

**DOI:** 10.1186/s12916-015-0535-9

**Published:** 2015-12-11

**Authors:** Erni J. Nelwan, Lenny L. Ekawati, Bagus Tjahjono, Rianto Setiabudy, Inge Sutanto, Krisin Chand, Tyas Ekasari, Dwi Djoko, Hasan Basri, W. Robert Taylor, Stephan Duparc, Decy Subekti, Iqbal Elyazar, Rintis Noviyanti, Herawati Sudoyo, J. Kevin Baird

**Affiliations:** Faculty of Medicine, University of Indonesia, Jalan Salemba Raya No. 6, Jakarta, 10430 Indonesia; Eijkman-Oxford Clinical Research Unit, Jalan Diponegoro No. 69, Jakarta, 10430 Indonesia; Health Services, Army of the Republic of Indonesia, Jalan Letjen Soetoyo, Jakarta, 13640 Indonesia; Medicines for Malaria Venture, Route de Pré-Bois 20, 1215 Meyrin, Switzerland; Eijkman Institute for Molecular Biology, Jalan Diponegoro No. 69, Jakarta, 10430 Indonesia; The Centre for Tropical Medicine, Nuffield Department of Medicine, University of Oxford, Old Road Campus, Roosevelt Drive, Oxford, OX3 7FZ UK

**Keywords:** Malaria, *Plasmodium vivax*, Relapse, Primaquine, Efficacy

## Abstract

**Background:**

Safety and efficacy of primaquine against repeated attacks of *Plasmodium vivax* depends upon co-administered blood schizontocidal therapy in radical cure. We assessed primaquine (PQ) as hypnozoitocide when administered with dihydroartemisinin-piperaquine (Eurartesim®, DHA-PP) or artesunate-pyronaridine (Pyramax®, AS-PYR) to affirm its good tolerability and efficacy. A third arm, artesunate followed by primaquine, was not intended as therapy for practice, but addressed a hypothesis concerning primaquine efficacy without co-administration of blood schizontocide.

**Methods:**

During March to July 2013, an open-label, randomized trial enrolled Indonesian soldiers with vivax malaria at Sragen, Central Java, after six months duty in malarious Papua, Indonesia. No malaria transmission occurred at the study site and *P. vivax* recurrences in the 12 months following therapy were classified as relapses. A historic relapse control derived from a cohort of soldiers who served in the same area of Papua was applied to estimate risk of relapse among randomized treatment groups. Those were: 1) AS followed 2d later by PQ (0.5 mg/kg daily for 14d); 2) co-formulated AS-PYR concurrent with the same regimen of PQ; or 3) co-formulated DHA-PP concurrent with the same regimen of PQ.

**Results:**

Among 532 soldiers, 219 had vivax malaria during the four months following repatriation to Java; 180 of these were otherwise healthy and G6PD-normal and enrolled in the trial. Subjects in all treatment groups tolerated the therapies well without untoward events and cleared parasitemia within three days. First relapse appeared at day 39 post-enrollment, and the last at day 270. Therapeutic efficacy of PQ against relapse by incidence density analysis was 92 % (95 %CI = 83–97 %), 94 %(95 %CI = 86–97 %), and 95 %(95 %CI = 88–98 %) when combined with AS, AS-PYR, or DHA-PP, respectively.

**Conclusions:**

This trial offers evidence of good tolerability and efficacy of PQ against *P. vivax* relapse when administered concurrently with DHA-PP or AS-PYR. These offer alternative partner drugs for radical cure with primaquine. The AS arm demonstrated efficacy with a total dose of 7 mg/kg PQ without concurrently administered blood schizontocide, another option when primaquine therapy is removed in time from the treatment of the acute malaria or applied presumptively without an attack.

**Trial registration:**

Current Controlled Trials ISRCTN82366390, assigned 20 March 2013.

## Background

*Plasmodium vivax* threatens nearly three billion people and sickens tens of millions annually [1]. Improper or delayed treatment may lead to severe and fatal outcomes associated with severe anemia, severe thrombocytopenia, respiratory distress, renal failure, hepatic dysfunction, coma, or shock [2, 3]. Unlike, *Plasmodium falciparum*, *P. vivax* places latent stages called hypnozoites in the liver after inoculation of mosquito-borne sporozoites. Hypnozoites later awaken and provoke renewed clinical attacks, each causing debilitating illness with risk of threatening complications and onward transmission.

In tropical Southeast Asia, *P. vivax* relapse behaviors resemble those in the Chesson strain from New Guinea [4, 5]. “Chesson-like” strains relapse quickly, repeatedly, and at approximately two-month intervals in almost all patients. In Thailand and Indonesia, relapse occurred among nearly 80 % of patients within two months [6, 7]. Among others treated for acute falciparum malaria, 51 % suffered *P. vivax* attacks within two months [6]. Relapse likely accounts for most attacks of vivax malaria where Chesson-like *P. vivax* occurs [8, 9]. Therapeutic success against hypnozoites may thus be appreciated as an important clinical and public health objective.

Radical cure of vivax malaria includes blood schizontocidal and hypnozoitocidal therapies. In 1952 the United States licensed the 8-aminoquinoline primaquine combined with the 4-aminoquinoline chloroquine for radical cure of *P. vivax* malaria. Primaquine remains the only option against relapse, although another 8-aminoquinoline, the investigational drug tafenoquine, approaches licensure [10]. Newer blood schizontocidal therapies must be combined with primaquine in radical cure because resistance to chloroquine by *P. vivax* often occurs, especially in Southeast Asia [11]. Resistant strains of *P. vivax* dominate across the Indonesian archipelago, and chloroquine was abandoned in favor of artemisinin-combined therapies (ACT) therapies over a decade ago [12].

Authoritative recommendations for the use of ACTs for treatment of acute *P. vivax* malaria cite good evidence of efficacy [13, 14]. However, no evidence yet attests to primaquine safety and efficacy when co-administered with most of those therapeutic options. The importance of such evidence has been highlighted by historic examples of drug-drug interactions (DDI) profoundly impacting both the safety and efficacy of 8-aminoquinolines [15]. Frontline therapy for acute *P. vivax* in Indonesia is the ACT dihydroartemisinin-piperaquine (DHA-PP) [12]. We reported good safety and efficacy of primaquine against relapse when administered 25 days after DHA-PP therapy, a delay imposed by the unexamined possibility of DDI [7]. Later studies affirmed no DDI issues with co-administered DHA-PP and primaquine [16]. The current study aimed to evaluate the safety and efficacy of primaquine administered concurrently with DHA-PP. We also evaluated primaquine co-administered with another ACT, artesunate-pyronaridine (AS-PYR) given recent evidence indicating a lack of significant pharmacokinetic interaction with primaquine [17]. Both of these blood schizontocidal therapies have very good efficacy against asexual blood stages of *P. vivax* [18–21].

The trial included a third treatment arm, artesunate [22] followed by primaquine. That arm was not intended as therapy for practice, but was an independent experimental arm testing the hypothesis that primaquine may require concurrent administration of partner blood schizontocides for good efficacy [15]. A trial in the 1950s demonstrated primaquine failure against relapse when administered after quinine rather than concurrently [23].

We recruited Indonesian soldiers exposed to malaria in eastern Indonesia after returning to a site in Central Java free of malaria transmission [24]. In addition to the distinct advantage of classifying recurrent malaria as relapses with a high degree of certainty, the infections were by the chloroquine-resistant and primaquine-tolerant Chesson-like *P. vivax* strains dominating eastern Indonesia [25].

## Methods

### Study design, setting and patient population

**Fig. 1:**
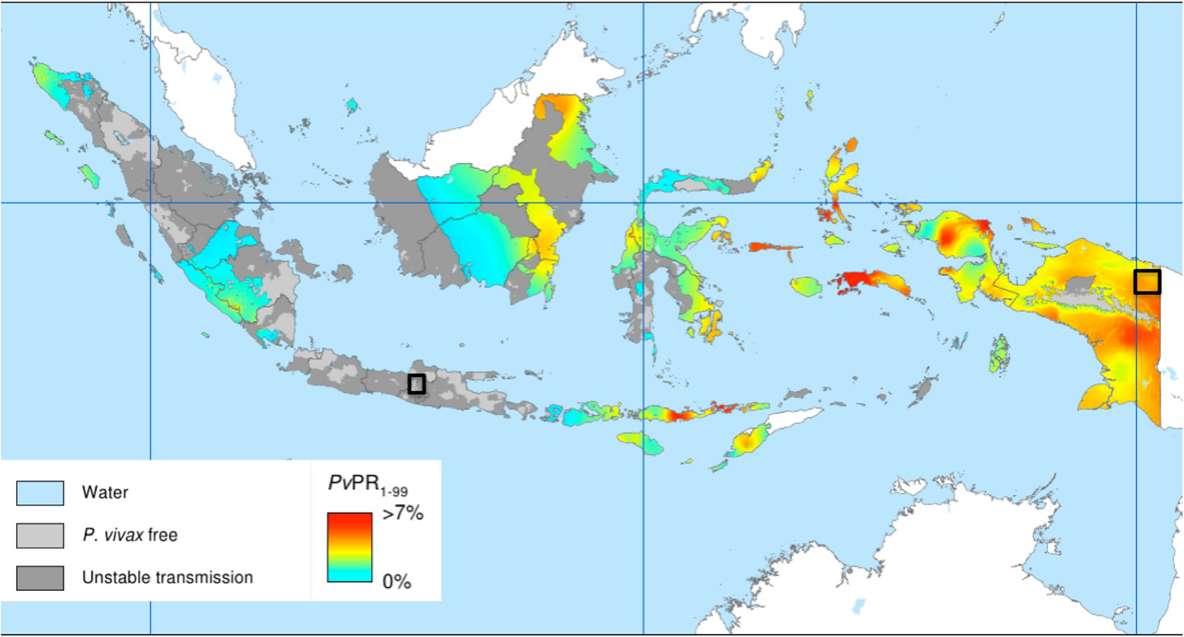
*Plasmodium vivax* in Indonesia and location of exposure of study battalion. Map of Indonesia illustrating predicted prevalence of *P. vivax* in 2010 published elsewhere [24]. Black box at *far right* indicates the area where both a prior study battalion [7] and the current one were exposed to risk of infection, and the black box at *center left* indicates location of the current study site in Central Java

A single center, randomized, open-label primaquine efficacy trial enrolled Indonesian soldiers with *P. vivax* malaria after returning from malarious Papua (Fig. 1) [24]. The study took place at an army base at Sragen, Central Java, between March 2013 and July 2014. The trial team established permanent residence, offices, clinic, pharmacy, and laboratory on that base.

The battalion identified for study returned to Java by ship, disembarking at Tanjung Mas at Semarang on 28 February 2013. Routine microscopic screening for malaria at portside revealed 67 of 536 soldiers had malaria (30 *P. falciparum*, 33 *P. vivax*, and 4 mixed infections). These soldiers received treatment according to national guidelines [12], but not primaquine because screening for glucose-6-phosphate dehydrogenase (G6PD) deficiency was not available. The soldiers departed on liberty to visit with families prior to returning to base. They had done so by 20 March 2013, when study familiarization began, including emphasis on the importance of voluntary consent. Enrollments commenced on 29 March 2013.

Soldiers on base faced no known risk of acquiring malaria locally. Sragen District (population 896,201 residents) occupies 942 km^2^ with an average density of 947 residents/km^2^ with 25 primary health care centers and 10 hospitals. During 2012–2013, authorities reported eight malaria cases, all imported. No known cases of locally acquired malaria were reported during 2013–2014. Reinfection at Sragen was highly improbable.

### Eligibility and randomization

Consenting soldiers with confirmed *P. vivax* were screened for eligibility by obtaining a medical history, and conducting physical and laboratory examinations that included electro-cardiogram (ECG; BTL-08, BTL Industries, Framingham, MA, USA; 12-lead, 50 mm/s, 10 mm/mV), complete blood counts (Coulter Ac-T; Beckman Coulter, Fullerton, CA, USA), full blood chemistry panel (Cobas-111, Roche, Rotkreuz, Switzerland), and G6PD deficiency screening (NADPH kit 203A, Trinity Biotech, Wicklow, Ireland). Eligible subjects had uncomplicated vivax malaria, were not under treatment for another illness, had not recently consumed antimalarials, and showed normal ECG, blood laboratory values, and G6PD activity by fluorescent spot testing (Trinity Biologics, Ireland).

The trial statistician block-allocated treatment assignments by varying blocking number at random [26]. A plain, consecutively numbered and sealed envelope prepared by the trial administrator contained therapy assignment and was opened in sequence as each eligible and consenting soldier enrolled. The study pharmacist supervised therapy and adherence to assigned treatment, including direct observation of each of the daily 14 doses of PQ.

### Study treatments

Groups received one of the following treatments under directly observed, signature-affirmed supervision by a member of the research team:

**AS + PQ** – 200 mg AS (Arsuamoon®; a 50-mg artesunate tablet co-packaged with an amodiaquine hydrochloride tablet [discarded]; Guilin Pharmaceuticals Co. Ltd., Shanghai, China) was administered, followed by a daily dose of 100 mg daily for six days. After a 48-hour pause for AS washout, PQ was administered with a daily dose of two tablets, each containing 15 mg PQ base for 14 days. As in all other groups, Sanofi Pharmaceuticals (PRIMAQUINE®, Primaquine phosphate tablets USP 26.3 mg, 15 mg equivalent base, Sanofi-Aventis Canada Inc. Laval, Quebec) provided the PQ administered.

**AS-PYR + PQ** – single daily dose of three tablets, each containing 60 mg AS and 180 mg of PYR provided as Pyramax® from Shin Poong Pharmaceuticals, Seoul, South Korea, for three days given concurrently with a daily dose of 30 mg PQ base for 14 days.

**DHA-PP + PQ** – a single daily dose of three tablets, each containing 40 mg DHA and 320 mg of PP base provided as Eurartesim® from Sigma-Tau Industrie Farmaceutiche Riunite S.p.A., Pomezia, Rome, Italy, for three days given concurrently with a daily dose of 30 mg PQ base for 14 days.

Subjects weighing >70 kg were treated as above except instead receiving 45-mg daily dose PQ (three tablets) for 14 days, five daily AS tablets instead of four, and four rather than three tablets of either AS, AS-PYR or DHA-PP daily. All subjects were offered a non-fatty carbohydrate snack prior to dosing to mitigate stomach upset caused by PQ [27]. Subjects were monitored for an hour and dosing repeated if vomiting occurred.

### Follow-up

Physical complaints and methemoglobin (Masimo Blood Oximeter, Masimo Corp., Irvine, CA, USA) were recorded daily during therapy. Routine blood film examinations occurred on days 3, 7, 14, 21, 28, 35, 42, 56, 63, 70, 84, 126, 140, 180, and 365 post-enrollment. Subjects reporting to the clinic with illness were examined the same day. Subjects positive for *P. vivax* received DHA-PP + PQ (0.5 mg/kg/day × 14 days) and were released from study after 28 days. Subjects not relapsing were followed for one year.

### Endpoints and definitions

Primary endpoint was incidence density (events/person-year) of first relapse by *P. vivax* in the year following radical cure. Patients reaching that endpoint contributed time at risk up to that event or withdrawal. Microscopic diagnoses of *P. vivax* were confirmed by another microscopist on site, and later by a blinded third microscopic read in Jakarta. Blood blots were collected onto Whatman FTA Classic™ filter paper for PCR confirmation of the diagnosis, and DNA extracted (QIAmp DNA Blood Mini™) before nested PCR *Plasmodium* genus-specific primers (Nest 1) and then with species-specific primers for Nest 2 as detailed elsewhere [28]. Secondary endpoints included frequencies of physical complaints, laboratory abnormalities, reported adverse events of grade 3 or higher during treatment, and serious adverse events possibly, probably, or definitely related to study treatment at any time during treatment or follow-up.

### Statistical considerations

We powered the trial for precision in independent estimates of PQ efficacy when combined with AS, AS-PYR, or DHA-PP. The precision estimate was derived iteratively through analysis of binomial distribution of variance around a predicted 98 % efficacy [7]. A sample size of 60 individuals per arm delivered the desired 3.5 % precision around the estimate of efficacy.

The primary endpoint of incidence density informed the estimate of efficacy derived from a relapse control. That control adjusts for geographic variability in relapse attack rates [4, 29]. We employed a relapse control cohort from a previous study [7] as relapse control in the current study. The cohort came from a prior battalion of study from Lumajang, East Java. They had been deployed to precisely the same locale in Papua as the current battalion of study at Sragen, Central Java. We reasoned the relapse attack rate would be similar. Therapeutic efficacy among treatment groups at Sragen was thus calculated using incidence density of relapse control (ID_AS-Lumajang_) against incidence density (ID) of recurrent *P. vivax* post-therapy among groups as follows:

$$ \left[\left(\mathrm{I}{\mathrm{D}}_{\mathrm{AS}\hbox{-} \mathrm{Lumajang}}\hbox{-} \mathrm{I}{\mathrm{D}}_{\mathrm{Sragen}\kern0.5em \mathrm{treatment}\kern0.5em \mathrm{groups}}\right)/\mathrm{I}{\mathrm{D}}_{\mathrm{AS}\hbox{-} \mathrm{Lumajang}}\right]\times 100 $$

The 95 % confidence interval (CI) for each of the three estimates of efficacy was calculated from the binomial distribution.

Incidence density of relapse represents the preferred mathematical treatment for events occurring over prolonged periods [30, 31]. Like efficacy of chemoprophylaxis against malaria [32], incidence density within groups was the number of *P. vivax* attacks divided by the sum of person-years at risk at end of study. Therapeutic efficacy expressed as proportion of subjects remaining free of recurrence after six months was included in this trial analysis for sake of reference to other hypnozoitocidal trials where reinfection possibly confounded recurrence rates [10].

### Ethics

Institutional ethics review boards of the Faculty of Medicine, University of Indonesia (ref.no. 13/H2.F1/ethic/2013) and the Centre for Tropical Medicine, Nuffield Department of Medicine, University of Oxford (ref.no.179-12) reviewed and approved a protocol detailing this trial. Indonesian food and drug regulatory authorities (*Badan Pengawasan Obat dan Makanan,* Jakarta; ref.no. PN.01.06.313.03.13.998) approved and monitored this trial.

## Results

### Patients and procedures

**Fig. 2:**
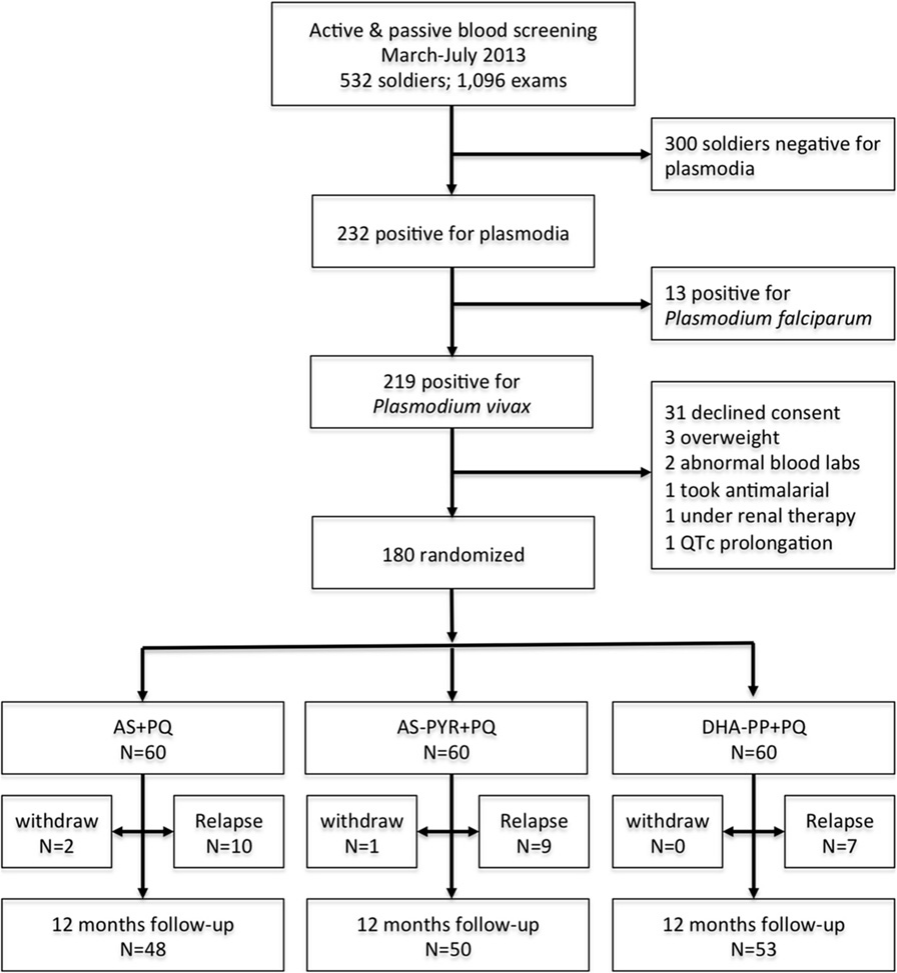
Study flow diagram

Figure 2 illustrates screening and enrollment during March-July 2013. Table 1 lists baseline demographic, laboratory and clinical characteristics among the randomized treatment groups. Subjects were 21–50 years old (mean 29–30 yr; P = 0.54), weighed 52–88 kg (means 67–71 kg; P = 0.045), and had parasitemias of *P. vivax* (confirmed by PCR) ranging from 16–9,488/μL (median 744-888/μL; P = 0.94). Fever (>37.5 °C) occurred in 22–30 % among groups (*P* = 0.72). No significant differences appeared between treatment groups in baseline vital signs or laboratory testing, except weight (P = 0.05). These same characteristics in the historic relapse control cohort are shown at the far right of Table 1 for reference without statistical comparison to randomized arms because no comparisons were made with this cohort. The non-randomized control subjects were essentially similar to the randomized treatment subjects in all demographic and clinical/laboratory respects.

**Table 1:** Baseline demographic, laboratory, and clinical features

Baseline feature	Randomized treatment assignment			p-value	Historic relapse control
	AS + PQ	AS-PYR + PQ	DHA-PP + PQ		AS alone
Subjects	60	60	60	--	41
Mean age (range)	29.4 (25–45)	28.6 (21–45)	29.5 (23–50)	0.54	27.7 (22–39)
Mean kg weight (range)	69 (55–87)	71 (54–88)	67 (52–88)	0.05	67 (52–90)
Tympanic °C					
Mean	37.1	37.3	37.2	0.37	36.7
Mean g/dL					
Hemoglobin	13.8	13.9	13.5	0.54	14.2
Mean %					
Methemoglobin	1.39	1.42	1.46	0.35	1.48
Median parasites/μL blood (range)	744 (16–7664)	888 (32–6016)	880 (16–9488)	0.94	1408 (32–15248)
WBCs × 10^3^/μL	7051	6886	6945	0.66	7700
Platelets × 10^3^/μL	171	166	161	0.75	167
Glucose mg/dL	106	108	103	0.73	108
Bilirubin mg/dL	1.03	0.97	1.04	0.69	1.08

*AS* artesunate, *PQ* primaquine, *PYR* pyronaridine, *DHA-PP* dihydroartemisinin-piperaquine

Table 2 lists the frequency, distribution, and character of illness among the three randomized arms at enrollment. Most subjects (127/180) were diagnosed with *P. vivax* after complaining of illness, but a minority was discovered during routine blood survey of the battalion (53/180). Illness was mild in most cases and dominated by headache, fever, chills, or muscle ache. A few subjects did suffer moderate illness with nausea or vomiting (12/180).

**Table 2:** Illness at enrollment

	Treatment assignment			Total
	AS + PQ	AS-PYR + PQ	DHA-PP + PQ	
Subjects	60	60	60	180
Diagnosis with illness	42	43	42	127
Diagnosis without illness	18	17	18	53
Headache	30	35	24	89
Fever	19	27	24	70
Muscle ache	12	14	6	32
Chills	12	8	10	30
Malaise	5	3	6	14
Nausea	1	6	1	8
Vomiting	1	0	3	4

*AS* artesunate, *PQ* primaquine, *PYR* pyronaridine, *DHA-PP* dihydroartemisinin-piperaquine

### Assessments to 63 days

Physical complaints resolved within several days. Nausea, vomiting, and headache occurred in fewer than 3 of 60 subjects among treatment arms. Adverse events of grade 3 or higher occurred among five, three, and one subject receiving AS + PQ, AS-PYR + PQ, and DHA-PP + PQ, respectively. None were definitely related to drug, and two were probably related to drug (vomiting with AS-PYR + PQ). Seven subjects were hospitalized during the study and each was classified as having experienced a serious adverse event unrelated to acute malaria or study drugs (dengue and typhoid fever, ureteric stone, nephrolithiasis, head trauma, fractured metacarpal, and gastroenteritis).

**Fig. 3:**
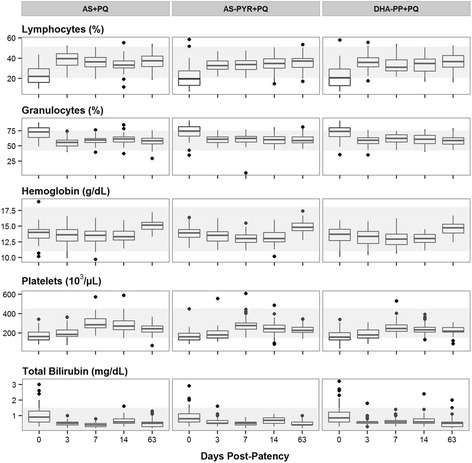
Abnormal blood values at enrollment. Graphs illustrating blood laboratory values that significantly changed during the course of treatments among groups. Each graph shows median (*horizontal line*), interquartile range (*box*), 1.5 times that range (*horizontal line*), and outlying observations (points). The lightly shaded area shows the range of normal values

Most blood chemistry and cell counts were normal at enrollment and did not deviate during follow-up. However, lymphocytes, platelets, granulocytes, total bilirubin, and hemoglobin were abnormal but returned to normal (Fig. 3): depressed lymphocytes within three days; depressed platelets at seven days; elevated granulocytes at three days; and elevated total bilirubin at three days. Depressed hemoglobin did not recover within 14 days, but did by day 63. All of these changes were attributed to acute malaria and recovery from it.

**Fig. 4:**
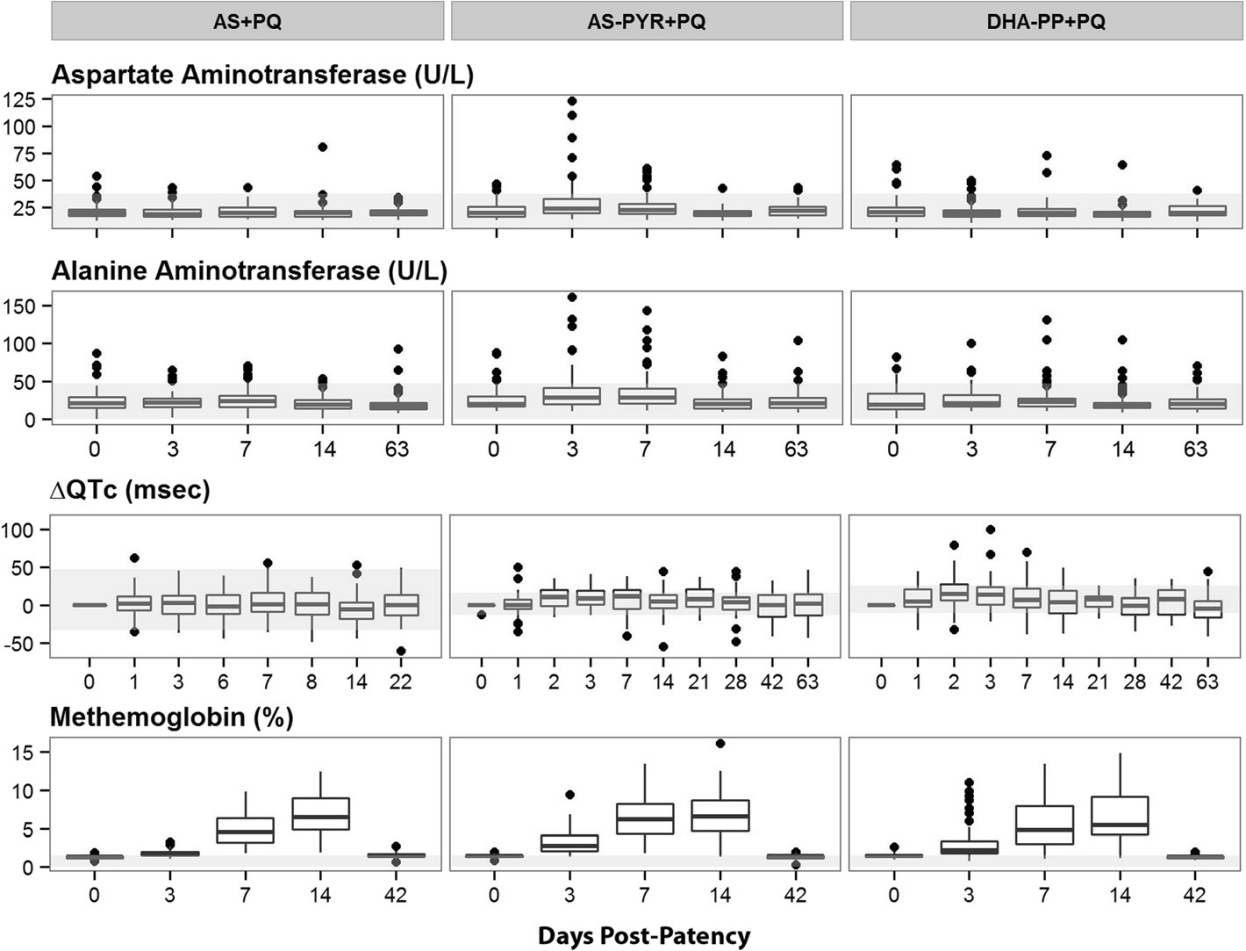
Changes in liver and blood chemistry and QTc values prior to and up to 63 days following treatment commencement of therapy. Graphs illustrating liver and blood laboratory values and QTc measurements that significantly altered after the commencement of therapy among groups. Each graph shows median (*horizontal line*), interquartile range (*box*), 1.5 times that range (*horizontal line*), and outlying observations (points). The lightly shaded area shows the range of normal values, except for ΔQTc which reflects the mean ΔQTc ± standard deviation. MetHb measurements for AS + PQ represent days post-dosing with PQ (commencing on day 9 post-patency). *MetHb* methemoglobin, *AS* artesunate, *PQ* primaquine

Liver and other blood chemistry values changed after initiating therapy (Fig. 4). Aspartate aminotransferase (AST) and alanine aminotransferase (ALT) levels became significantly elevated at days 3 and 7 in the group receiving AS-PYR (P = 0.00001 and P = 0.0032), but not with AS or DHA-PP for either AST or ALT (P = 0.30-0.86). The AS-PYR median AST and ALT values, although elevated above baseline, remained within normal limits, except for a minority of subjects (Fig. 4). Those AST and ALT median values in the AS-PYR group each returned to baseline values by day 14, and total bilirubin declined normally (by day 7) in this group. No detectable changes to QTc interval occurred during therapy with AS (day 1 vs. day 3, P = 0.92) (Fig. 4). Modest QTc elongation occurred during treatment with AS-PYR + PQ and DHA-PP + PQ: day 1 vs. day 2, P = 0.0083 and P = 0.0285, respectively (Fig. 4). The effect lingered to day 7 with AS-PYR + PQ (P = 0.0074) but not to day 14 (P = 0.252). The effect with DHA-PP + PQ was absent by day 7 (P = 0.82). Among all treatment groups, methemoglobin levels increased from a baseline of about 1 % to 5–6 % within ten days, and became normal within two weeks post-dosing of PQ (Fig. 4).

### Relapse control

**Fig. 5:**
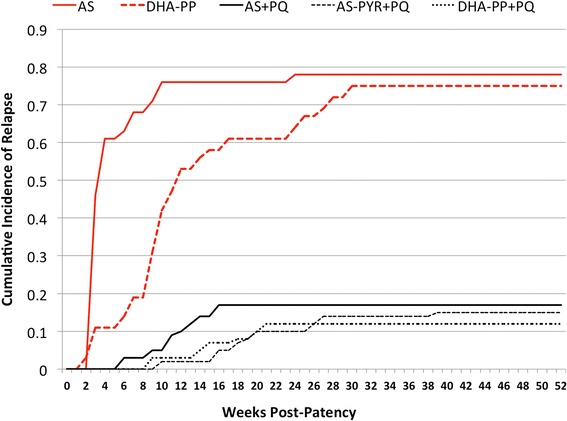
Relapse attack among groups during year after therapy. Relapse timing and extent among treatment groups (AS + PQ, *solid black*; AS-PYR + PQ, *black dashed*; DHA-PP + PQ, *black dotted*) in the current trial. The *upper solid red line* (AS) represents the relapse control group used as the denominator in estimating efficacy, taken from a prior trial in soldiers deployed to the same region [7]. The other *upper red line that is dashed* (ACT only) represents relapse events among 39 soldiers with vivax malaria at Sragen who declined participation and were treated with DHA-PP but without primaquine. *AS* artesunate, *PQ* primaquine, *PYR* pyronaridine, *DHA-PP* dihydroartemisinin-piperaquin

Figure 5 illustrates weekly cumulative incidence of relapse among randomized treatment arms during 52 weeks. The graph also illustrates relapse among 41 soldiers enrolled in another study and randomized to AS only (no PQ), the historic relapse control in the current study. Thirty-six soldiers in the current trial at Sragen were diagnosed with vivax malaria but not enrolled in the current study and received DHA-PP only (see Fig. 2). Many later reported to the study clinic with confirmed relapse. These observational findings allowed an independent assessment of the per-protocol relapse historic control as a surrogate of relapse attack in this trial: incidence density 2.7/person-yr vs. 2.5/person-yr (P = 0.9); one-year cumulative incidence of relapse 78 % vs. 75 % (P = 0.79), respectively.

### Therapeutic efficacy

All subjects cleared asexual parasitemia by day 3. Most subjects were gametocytemic at enrollment (121/160), representing 60 %, 62 %, and 70 % rates among AS + PQ, AS + PYR, and DHA-PP groups, respectively (P = 0.342). Gametocytemia occurred more frequently among symptomatic (98/127, 77 %) compared to asymptomatic (23/53, 43 %) subjects; OR = 4.4 (95 %CI = 2.2-8.8;P < 0.0001). The initial geometric mean (range) per μL of 128(16–1232), 80(16–1200), and 64(16–1200) among those respective groups (P = 0.287) vanished within 24 hr of initiating therapy except in one, one, and two subjects. All were free of microscopically patent gametocytemia by day 3. Table 3 summarizes primary endpoint analyses estimating efficacy of PQ against relapse when combined with AS, AS-PYR, or DHA-PP. The groups of 60 subjects experienced ten, nine, and seven relapses, respectively, with only three subjects being lost to follow-up. Relapses occurred between day 39 and 270, with median day of relapse among groups at 78, 128, and 104, respectively. Therapeutic efficacy by incidence density was 92 %, 94 %, and 95 %, respectively, with 95 % confidence intervals between 83 %-98 %. Subjects >70 kg were not more likely to relapse (OR = 0.92; 95%CI = 0.4-2.3; P = 0.99). Estimates of efficacy by proportion of subjects remaining free of recurrence at six months, a secondary analysis for the sake of reference to other studies possibly confounded by reinfection, were 83 %, 88 %, and 88 % for AS + QN, AS + PYR, and DHA-PP, respectively.

**Table 3:** Primary endpoint analysis and therapeutic efficacy

	Treatment assignment			Relapse control
	AS + PQ	AS-PYR + PQ	DHA-PP + PQ	
Subjects	60	60	60	41
Withdrawals	2	1	0	0
Relapses	10	9	7	32
Efficacy by incidence density				
Person-years at risk	50.17	53.62	55.00	11.83
Relapse incidence density (attacks/person-yr)	0.20	0.17	0.13	2.71
Efficacy (%) against relapse (95 % CI)	92.2 (83.4 – 96.6)	93.5 (85.7 – 97.3)	95.0 (88.3 – 98.2)	na
Efficacy by proportion relapse-free at 6 months				
Relapses within 6 months	10	7	7	na
% free of recurrence at 6 Months	83	88	88	na

*AS* artesunate, *PQ* primaquine, *PYR* pyronaridine, *DHA-PP* dihydroartemisinin-piperaquine

## Discussion

This randomized trial of 0.5 mg/kg PQ daily for 14 days concurrent with AS-PYR or DHA-PP for radical cure of vivax malaria demonstrated good safety, tolerability, and efficacy for these options. All subjects cleared parasitemia by day 3 and none recurred before day 39, affirming good efficacy of AS-PYR and DHA-PP against the asexual blood stage parasites [18–21]. Initially high rates of gametocytemia rapidly cleared (<72 hr). Relapse occurred among 9/60 (15 %) and 7/60 (12 %) subjects assigned AS-PYR or DHA-PP, respectively. Incidence density was applied as the preferred mathematical treatment to derive estimates of therapeutic efficacy involving prolonged periods of follow-up [30–32]. We estimated the efficacy of radical cure by AS-PYR + PQ and DHA-PP + PQ at 94 % and 95 %, respectively (Table 3), each with good tolerability.

Drug-related changes occurred among subjects. The significant elevation of AST/ALT in a minority of subjects receiving AS-PYR (5/60) has been reported in other studies [33]. QTc elongation appeared in subjects taking AS-PYR and DHA-PP, but not AS + PQ (Fig. 4). These effects are known with those compounds and were not exacerbated by primaquine (daily 0.24 mg/kg dosing) in DDI studies in healthy volunteers [16, 17]. The data in the current trial are consistent with those observations. Consumption of a carbohydrate snack immediately prior to each primaquine dose very likely explains the relatively very low rates of vomiting despite high dose primaquine [27].

The AS + PQ treatment in the current trial tested the hypothesis that PQ requires concurrent blood schizontocidal therapy for good efficacy against hypnozoites. In 1955, Alving et al. [23] demonstrated inferior efficacy of PQ administered after quinine therapy (15/19 relapsed) relative to concurrent administration of same therapies at the same doses (2/19 relapsed) among those randomized groups. We administered AS first, then commenced PQ after a two day washout; 10/60 subjects relapsed, but no more frequently than among the other two treatment arms (P > 0.5). A higher total dose of PQ in the current trial (7 mg/kg) compared to Alving et al. [23] (3.5 mg/kg) likely explains the good efficacy of PQ despite otherwise similar treatment (artesunate and quinine are each efficacious against asexual blood stages and eliminated within 24 hr of dosing). PQ at the higher dose may thus be administered after treating the acute attack with assurance of good efficacy. This offers a therapeutic option when concurrent administration is either inconvenient or contraindicated. Primaquine at the 7 mg/kg total dose may thus commence at any point after therapy of acute malaria, or presumptively without an acute attack (as with post-travel presumptive anti-relapse therapy) with assurance of good efficacy.

PQ is prescribed at a total dose of either 3.5 or 7.0 mg/kg over 14 days, depending upon geographic origin of infection [34]. The New Guinea Chesson strain responded to daily 7.0 mg/kg, but not 3.5 mg/kg [35]. Studies confirm the tolerance of *P. vivax* strains from tropical Southeast Asia and New Guinea to the lower dose [25, 35, 36]. Despite applying the higher dose under direct supervision, 14 % (26/180) of our subjects relapsed. Possible explanations include hypnozoite resistance to PQ [37], and cytochrome P-450 2D6 (CYP2D6) polymorphisms impeding conversion of PQ to active metabolites [38, 39]. We are now examining CYP2D6 polymorphisms among the therapeutic failures in this trial and anticipate reporting these findings in the near future. Uncertainty regarding the basis of relapse despite high dose PQ therapy is a limitation of this study.

In addition to the primary incidence density endpoint analysis, Table 3 also shows crude estimates of efficacy as expressed by proportion of subjects remaining free of recurrent parasitemia after six months. This endpoint has been used in trials of hypnozoitocides and meta-analyses of them. Those trials were conducted where reinfection among treatment groups could not be excluded, or where variable risks and timing of natural relapse was not controlled in trial design. In other words, rather than report efficacy against relapse, those trials simply compare the effect of hypnozoitocidal therapy upon rates of recurrence from any source of bloodstream infection. In the trial reported here, by virtue of near certainty regarding the absence of confounding by recrudescence (wholly effective blood schizontocides) or reinfection (absence of risk at the study site), along with a relapse control arm (risk and rate of relapse without primaquine therapy), we were able to report therapeutic efficacy of primaquine against relapse using incidence density risk analysis.

This trial provided robust estimates of PQ efficacy against relapse when combined with modern therapies. The findings of good safety, tolerability and efficacy in this trial reassures, but does not address the serious problem of PQ effectiveness. PQ often fails because it is: 1) unavailable or prohibited [40]; 2) rarely prescribed [41]; 3) withheld from patients who may be pregnant, <1 yr of age, or deficient in G6PD [34]; 4) not offered to patients of unknown G6PD status [42]; 5) not completed by the patient [43]; or 6) disabled by CYP2D6 polymorphisms [37, 38, 44]. The sum of these potential pitfalls deeply erodes PQ effectiveness in routine practice in the often-impoverished endemic areas of the tropics. Good efficacy is the starting point for then addressing a range of barriers to the good effectiveness that realizes maximum clinical and public health impacts.

## Conclusions

Patients with *P. vivax* receiving AS-PYR + PQ or DHA-PP + PQ enjoyed good tolerability, safety and efficacy of these regimens. AS-PYR and DHA-PP may thus be considered validated options to chloroquine for radical cure of *P. vivax* with PQ. Further, PQ at 7 mg/kg total dose does not require concurrent administration of blood schizontocide for good efficacy. PQ may thus follow therapy with untried blood schizontocides for either therapy or chemoprophylaxis with the expectation of good efficacy.
